# Supplementary material for: Pyruvate Dehydrogenase Kinase 1 inhibition mediated oxidative phosphorylation enhancement in cartilage promotes osteoarthritis progression
Source: BMC Musculoskelet Disord. 2023 Jul 20;24:597. doi: 10.1186/s12891-023-06585-6 (PMC10357736; doi:10.1186/s12891-023-06585-6)
Supplement: Supplementary file 1 — Additional file 1. [file 12891_2023_6585_MOESM1_ESM.zip › KEGG.Gsea.1653622432464/KEGG_SNARE_INTERACTIONS_IN_VESICULAR_TRANSPORT.html]

Details for gene set KEGG\_SNARE\_INTERACTIONS\_IN\_VESICULAR\_TRANSPORT[GSEA]

|  || Dataset | 55457\_collapsed\_to\_symbols.cls.cls #OA\_versus\_normal.cls.cls #OA\_versus\_normal\_repos |
| Phenotype | cls.cls#OA\_versus\_normal\_repos |
| Upregulated in class | OA |
| GeneSet | KEGG\_SNARE\_INTERACTIONS\_IN\_VESICULAR\_TRANSPORT |
| Enrichment Score (ES) | 0.28927922 |
| Normalized Enrichment Score (NES) | 1.0594753 |
| Nominal p-value | 0.35034803 |
| FDR q-value | 0.5123507 |
| FWER p-Value | 1.0 |
Table: GSEA Results Summary

  

Fig 1: Enrichment plot: KEGG\_SNARE\_INTERACTIONS\_IN\_VESICULAR\_TRANSPORT      
 Profile of the Running ES Score & Positions of GeneSet Members on the Rank Ordered List

  

| SYMBOL | TITLE | RANK IN GENE LIST | RANK METRIC SCORE | RUNNING ES | CORE ENRICHMENT || 1 | STX8 | syntaxin 8 [Source:HGNC Symbol;Acc:HGNC:11443] | 204 | 0.815 | 0.0748 | Yes |
| 2 | VTI1B | vesicle transport through interaction with t-SNAREs 1B [Source:HGNC Symbol;Acc:HGNC:17793] | 336 | 0.732 | 0.1460 | Yes |
| 3 | VAMP8 | vesicle associated membrane protein 8 [Source:HGNC Symbol;Acc:HGNC:12647] | 676 | 0.599 | 0.1862 | Yes |
| 4 | BNIP1 | BCL2 interacting protein 1 [Source:HGNC Symbol;Acc:HGNC:1082] | 824 | 0.563 | 0.2374 | Yes |
| 5 | BET1 | Bet1 golgi vesicular membrane trafficking protein [Source:HGNC Symbol;Acc:HGNC:14562] | 1223 | 0.471 | 0.2589 | Yes |
| 6 | USE1 | unconventional SNARE in the ER 1 [Source:HGNC Symbol;Acc:HGNC:30882] | 1723 | 0.395 | 0.2640 | Yes |
| 7 | STX6 | syntaxin 6 [Source:HGNC Symbol;Acc:HGNC:11441] | 2863 | 0.264 | 0.2048 | Yes |
| 8 | SNAP29 | synaptosome associated protein 29 [Source:HGNC Symbol;Acc:HGNC:11133] | 2885 | 0.262 | 0.2323 | Yes |
| 9 | STX12 | syntaxin 12 [Source:HGNC Symbol;Acc:HGNC:11430] | 2886 | 0.262 | 0.2614 | Yes |
| 10 | STX2 | syntaxin 2 [Source:HGNC Symbol;Acc:HGNC:3403] | 2901 | 0.260 | 0.2893 | Yes |
| 11 | VAMP7 | vesicle associated membrane protein 7 [Source:HGNC Symbol;Acc:HGNC:11486] | 3380 | 0.214 | 0.2759 | No |
| 12 | VAMP5 | vesicle associated membrane protein 5 [Source:HGNC Symbol;Acc:HGNC:12646] | 3935 | 0.162 | 0.2508 | No |
| 13 | STX7 | syntaxin 7 [Source:HGNC Symbol;Acc:HGNC:11442] | 4967 | 0.075 | 0.1790 | No |
| 14 | STX18 | syntaxin 18 [Source:HGNC Symbol;Acc:HGNC:15942] | 4968 | 0.075 | 0.1873 | No |
| 15 | VAMP3 | vesicle associated membrane protein 3 [Source:HGNC Symbol;Acc:HGNC:12644] | 5028 | 0.071 | 0.1906 | No |
| 16 | GOSR2 | golgi SNAP receptor complex member 2 [Source:HGNC Symbol;Acc:HGNC:4431] | 5037 | 0.070 | 0.1977 | No |
| 17 | STX10 | syntaxin 10 [Source:HGNC Symbol;Acc:HGNC:11428] | 5726 | 0.013 | 0.1458 | No |
| 18 | YKT6 | YKT6 v-SNARE homolog [Source:HGNC Symbol;Acc:HGNC:16959] | 5800 | 0.008 | 0.1410 | No |
| 19 | SNAP25 | synaptosome associated protein 25 [Source:HGNC Symbol;Acc:HGNC:11132] | 5872 | 0.004 | 0.1359 | No |
| 20 | GOSR1 | golgi SNAP receptor complex member 1 [Source:HGNC Symbol;Acc:HGNC:4430] | 6166 | -0.018 | 0.1152 | No |
| 21 | STX5 | syntaxin 5 [Source:HGNC Symbol;Acc:HGNC:11440] | 6932 | -0.074 | 0.0640 | No |
| 22 | VAMP1 | vesicle associated membrane protein 1 [Source:HGNC Symbol;Acc:HGNC:12642] | 6971 | -0.078 | 0.0697 | No |
| 23 | SNAP23 | synaptosome associated protein 23 [Source:HGNC Symbol;Acc:HGNC:11131] | 7547 | -0.122 | 0.0386 | No |
| 24 | VAMP4 | vesicle associated membrane protein 4 [Source:HGNC Symbol;Acc:HGNC:12645] | 7608 | -0.128 | 0.0481 | No |
| 25 | STX17 | syntaxin 17 [Source:HGNC Symbol;Acc:HGNC:11432] | 8466 | -0.191 | 0.0028 | No |
| 26 | SEC22B | "SEC22 homolog B, vesicle trafficking protein [Source:HGNC Symbol;Acc:HGNC:10700]" | 8614 | -0.202 | 0.0138 | No |
| 27 | STX1A | syntaxin 1A [Source:HGNC Symbol;Acc:HGNC:11433] | 8648 | -0.204 | 0.0339 | No |
| 28 | VAMP2 | vesicle associated membrane protein 2 [Source:HGNC Symbol;Acc:HGNC:12643] | 8932 | -0.226 | 0.0371 | No |
| 29 | STX4 | syntaxin 4 [Source:HGNC Symbol;Acc:HGNC:11439] | 9222 | -0.250 | 0.0425 | No |
| 30 | STX3 | syntaxin 3 [Source:HGNC Symbol;Acc:HGNC:11438] | 10567 | -0.380 | -0.0197 | No |
| 31 | STX11 | syntaxin 11 [Source:HGNC Symbol;Acc:HGNC:11429] | 11711 | -0.535 | -0.0490 | No |
| 32 | BET1L | Bet1 golgi vesicular membrane trafficking protein like [Source:HGNC Symbol;Acc:HGNC:19348] | 12084 | -0.615 | -0.0095 | No |
| 33 | STX16 | syntaxin 16 [Source:HGNC Symbol;Acc:HGNC:11431] | 12235 | -0.655 | 0.0517 | No |
Table: GSEA details [plain text format]

  

Fig 2: KEGG\_SNARE\_INTERACTIONS\_IN\_VESICULAR\_TRANSPORT      
 Blue-Pink O' Gram in the Space of the Analyzed GeneSet

  

Fig 3: KEGG\_SNARE\_INTERACTIONS\_IN\_VESICULAR\_TRANSPORT: Random ES distribution      
 Gene set null distribution of ES for **KEGG\_SNARE\_INTERACTIONS\_IN\_VESICULAR\_TRANSPORT**
